# Coral Crisis: Palytoxin-Induced Keratoconjunctivitis in Marine Experts

**DOI:** 10.7759/cureus.72538

**Published:** 2024-10-28

**Authors:** Nikolas P Foresteire, Luis Cabrera, Cory Howard, Alexandra Craen, Ori Gat

**Affiliations:** 1 Emergency Medicine, HCA Florida/University of South Florida Morsani College of Medicine Graduate Medical Education Consortium at Brandon Hospital, Brandon, USA

**Keywords:** coral toxin, keratoconjunctivitis, marine experts, marine toxin, medical toxicology, palytoxin, poison control, red organ coral, toxicology, toxin-induced keratoconjunctivitis

## Abstract

Palytoxin (PTX or PLTX), a potent and rare marine toxin derived from certain coral species, poses significant risks to human health upon exposure, including the development of acute respiratory distress syndrome. We present a case report detailing the experiences of two expert marine biologists who inadvertently came into contact with palytoxin while handling marine organisms in a controlled environment. Shortly after exposure, both biologists developed severe keratoconjunctivitis, characterized by acute inflammation of the conjunctiva and cornea. Clinical manifestations included intense ocular pain, photophobia, conjunctival injection, and corneal epithelial defects, with symptoms progressing rapidly post exposure. Initial management involved immediate irrigation with a saline solution, followed by topical corticosteroids and lubricants to alleviate inflammation and promote corneal healing. Both individuals responded favorably to treatment, with resolution of symptoms within several days. This report underscores the importance of stringent adherence to safety protocols and awareness among marine biologists regarding the risks associated with palytoxin exposure, emphasizing the need for prompt recognition and appropriate management of ocular manifestations to minimize potential long-term sequelae.

## Introduction

Palytoxin (PTX or PLTX) is an intense, vasoconstricting, heat-stable, and potent marine toxin found in the dinoflagellate genus of Ostreopsis [1]. It has been isolated in many coral species, including Palythoa and zoanthid coral. It is known to be one of the most toxic non-peptide substances, posing a significant health risk to humans through various routes of exposure, including dermal contact, inhalation, and ingestion [1,2]. While documented cases of palytoxin poisoning primarily involve aquarium hobbyists and professionals handling marine organisms, instances of accidental exposure among seasoned marine biologists are rare but may provide valuable insights into the toxin's effects, management, and profound impact on human health.

This case report details the experiences of two expert marine biologists who encountered palytoxin during the routine handling of coral specimens in a controlled setting. Despite their extensive knowledge and adherence to safety protocols, both biologists developed severe keratoconjunctivitis shortly after exposure, underscoring the toxin's ability to cause debilitating ocular effects. The rapid onset and progression of symptoms highlight the acute nature of palytoxin toxicity and emphasize the importance of prompt recognition and appropriate management strategies to mitigate its potentially serious consequences. The clinical course of their illness was characterized by acute onset of ocular pain, photophobia, conjunctival inflammation, and corneal epithelial damage, highlighting the toxin's rapid and debilitating effects on ocular tissues [2-4].

Through this report, we aim to contribute to the existing literature on palytoxin toxicity by providing a detailed clinical course, management approach, and outcomes observed in these two cases. Additionally, we discuss preventive measures and safety protocols that can aid in minimizing the risk of palytoxin exposure among marine biologists and other professionals working with marine organisms in both research and recreational settings.

## Case presentation

Patient 1

A 56-year-old male marine biologist presented to the emergency department approximately eight hours after exposure to red organ coral. While relocating a live aquarium fish tank without personal protective equipment (PPE), he experienced bilateral eye pruritus, which progressed to blurry vision and bilateral eye pain. Despite immediate decontamination measures, including eye washing and showering, his symptoms persisted, prompting him to seek medical attention. He denied any systemic symptoms such as difficulty breathing, drooling, confusion, headaches, numbness, tingling, chest pain, nausea, vomiting, or diarrhea.

The patient's vital signs revealed a heart rate of 88 bpm, respiratory rate of 16 breaths/min, pulse oximetry of 97% on room air, hypertensive blood pressure of 178/97 mm Hg, and temperature of 36.6°C.

Physical Examination

**Figure 1:**
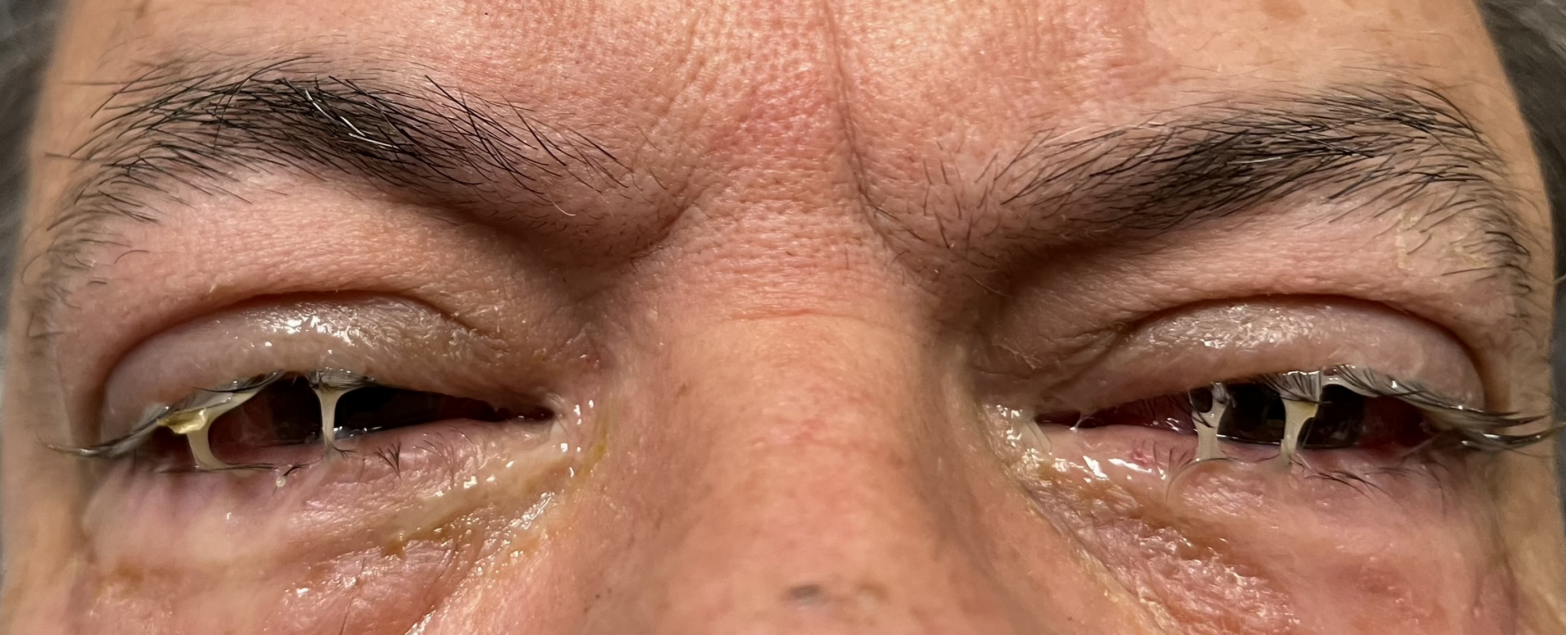
Palytoxin-induced keratoconjunctivitis in patient 1.

Bilateral keratoconjunctivitis with subconjunctival erythema (Figure 1), mild decreased visual acuity due to pain and excessive lacrimation, and intact painless extraocular movements were noted. Fluorescein examination showed no corneal ulcerations, abrasions, or foreign bodies. Confrontational visual acuity was intact bilaterally.

Treatment Plan

Based on the patient's expert knowledge and suspicion of palytoxin exposure, he was treated in the emergency department with tetracaine eye drops for pain control, proparacaine hydrochloride (HCL) ophthalmic drops, prednisolone acetate ophthalmic drops, moxifloxacin HCL ophthalmic drops, and 12 mg Decadron orally (PO). He was discharged home on moxifloxacin HCL ophthalmic drops, prednisolone acetate ophthalmic drops, and doxycycline hyclate PO.

Outcome

After approximately three hours of observation, the patient showed significant improvement in chemosis and pain. He was discharged home with an ophthalmology follow-up.

Patient 2

A 56-year-old male marine biologist presented to the emergency department after assisting in moving a fish tank containing coral known for palytoxin without utilizing any PPE. He reported intense lacrimation, eye pain, conjunctival erythema, and periorbital edema. Despite thorough eye flushing, his symptoms intensified, leading to emergency evaluation. He denied any foreign bodies, UV exposure, or systemic symptoms.

The patient's vital signs revealed a heart rate of 76 bpm, respiratory rate of 16 breaths/min, pulse oximetry of 96% on room air, hypertensive blood pressure of 207/120 mm Hg, and temperature of 37°C.

Physical Examination

**Figure 2:**
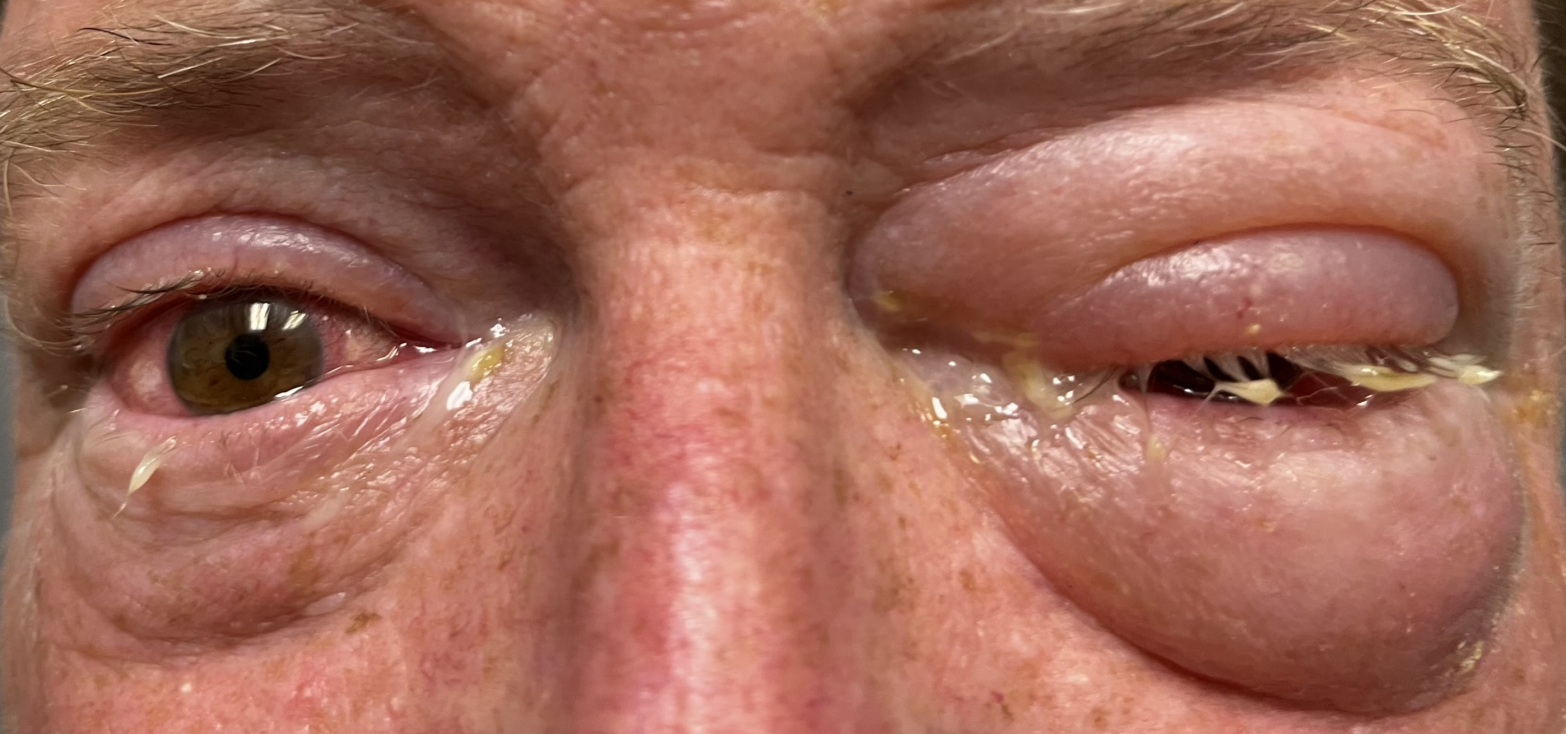
Palytoxin-induced keratoconjunctivitis with periorbital edema in patient 2.

Severe left periorbital edema and moderate chemosis in the left eye, mild chemosis and periorbital edema in the left eye, increased lacrimation, and purulence from the lower eyelids bilaterally were noted (Figure 2). Painless extraocular movements were intact. Fluorescein examination showed no corneal ulcerations, abrasions, or foreign bodies. Confrontational visual acuity was intact bilaterally.

Treatment Plan

The patient received dexamethasone sodium 12 mg PO, moxifloxacin ophthalmic drops, prednisolone acetate ophthalmic drops, and tetracaine HCL ophthalmic drops in the emergency department. He was discharged home on moxifloxacin HCL ophthalmic drops, prednisolone acetate ophthalmic drops, and doxycycline hyclate PO.

Outcome

After approximately three hours of observation, the patient showed significant improvement in chemosis and pain. He was discharged home with an ophthalmology follow-up.

## Discussion

**Figure 3:**
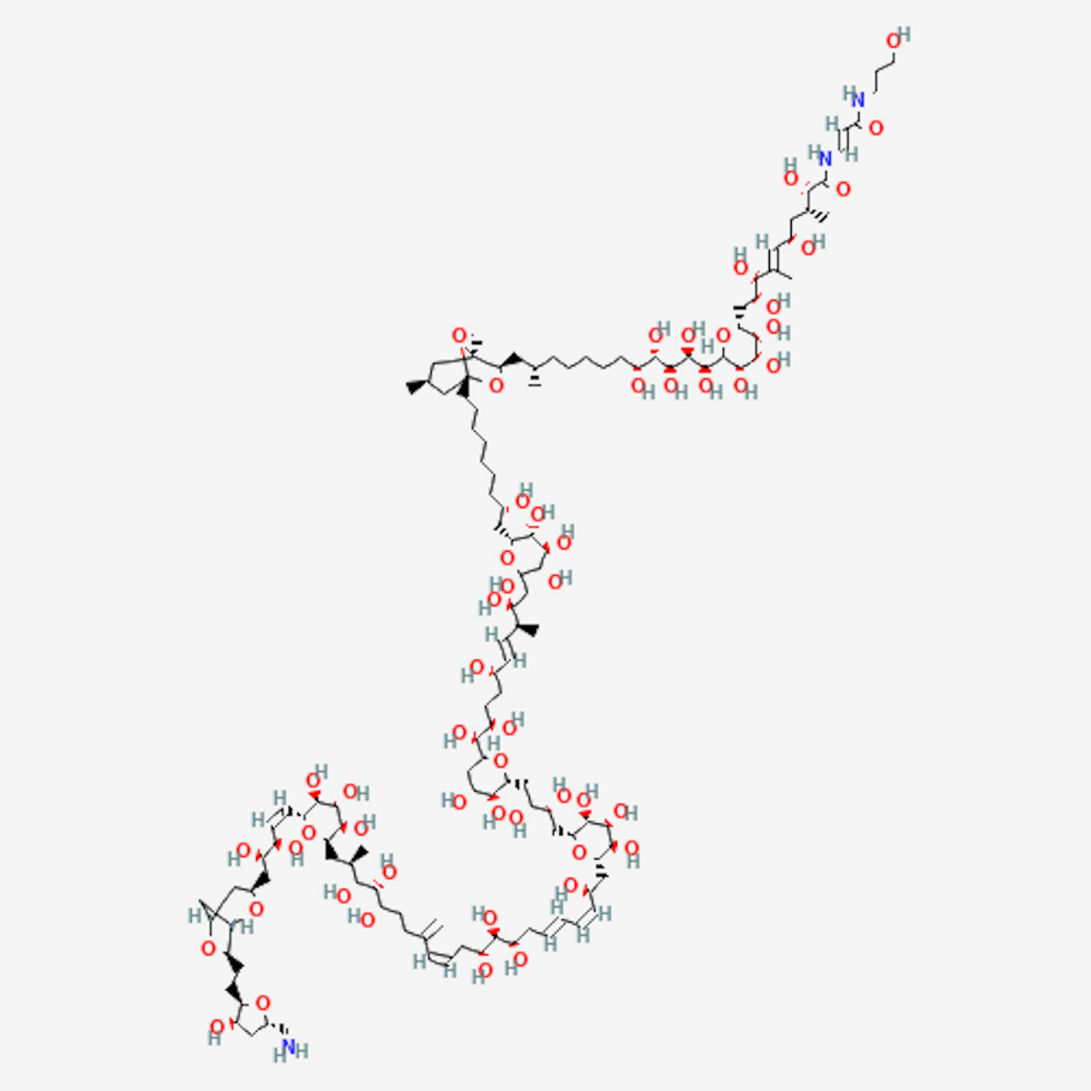
2D chemical structure of palytoxin (PLX). Source: National Center for Biotechnology Information (2024). PubChem Compound Summary for CID 11105289, Palytoxin. Retrieved October 1, 2024 from https://pubchem.ncbi.nlm.nih.gov/compound/11105289#section=2D-Structure [7].

Palytoxin (PTX or PLTX) is an intense, vasoconstricting, heat-stable, and potent marine toxin found in the dinoflagellate genus of Ostreopsis. It has been isolated in many coral species, including Palythoa and zoanthid coral. It is known to be one of the most toxic non-peptide substances. Outlined in Figure 3, palytoxin has a large molecular structure with 64-isolate chiral centers making it both very lipophilic, hydrophilic, and largely carbon-chained [1]. It is one of the longest carbon-containing chains of all naturally occurring molecules [2-7].

Pathophysiology

Palytoxin has been proposed to disrupt cellular ion gradients by utilizing ATP hydrolysis to inhibit the function of the Na+/K+-ATPase channel, open the ion pump, and promote massive calcium cytosol release at the sub-cellular level [1]. It is thought that this calcium ion channel binding leads to the contraction of cytoskeleton elements including actin, which commonly results in muscular contraction resulting in rhabdomyolysis, myokymia, cardiac arrhythmias, renal failure, cyanosis, and acute respiratory distress [1]. As a result of disrupting this vital mitochondrial pump, it can lead to cell lysis, inflammation, and resultant significant tissue damage [3]. While systemic effects of palytoxin are well-documented, ocular toxicity, particularly keratoconjunctivitis, remains relatively underreported. Palytoxin ocular exposure can result in a severe inflammatory response, leading to keratoconjunctivitis characterized by conjunctival erythema (redness), chemosis (swelling), lacrimation (increased tear production), and pain. The molecular interactions described above likely result in localized corneal epithelial death, disruption, ulceration of the stromal layer, and inflammation. As the eye attempts to heal, corneal myofibroblasts containing alpha-actin may be further disrupted by PTX, causing continued tissue death, delayed healing, and increased long-term ocular sequelae, including proposed peripheral degradation of collagen, keratocytes, and proteoglycans leading to corneal thinning [3-6].

Clinical presentation

Both patients in this report presented with acute onset of bilateral eye pruritus (itching), progressing to pain, blurred vision, and excessive lacrimation shortly after exposure to red organ coral. We propose that the absence of systemic symptoms such as respiratory distress, neurological deficits, or gastrointestinal disturbances, and the specific ocular manifestations, aligned with localized palytoxin exposure. These findings underscore the need for healthcare providers to recognize ocular symptoms as potential indicators of palytoxin toxicity, especially in individuals handling marine corals.

Management

Palytoxin-induced keratoconjunctivitis management focuses on prompt identification, decontamination, and symptomatic relief through supportive measures. Both patients underwent immediate ocular irrigation and decontamination, a crucial step in reducing toxin load and preventing further damage. The use of topical antibiotics (moxifloxacin HCL) helped prevent secondary bacterial infections, while corticosteroids (prednisolone acetate) mitigated inflammation. Pain control was effectively achieved with tetracaine and proparacaine HCL ophthalmic drops. Systemic corticosteroids (Decadron and dexamethasone sodium) were also administered to further control inflammation, thus providing further symptomatic care.

The rapid improvement in both patients' symptoms highlights the efficaciousness of this treatment regimen. However, these cases also emphasize the need for extended observation and follow-up to monitor for potential delayed complications or persistent symptoms.

## Conclusions

These two reported cases of simultaneous palytoxin-induced keratoconjunctivitis highlight the ocular risks associated with handling red pipe organ coral. They also underscore the importance of immediate and appropriate medical intervention. Early recognition and treatment are crucial to prevent long-term ocular damage. Preventive measures, including the use of PPE and proper training, are essential to safeguard against such toxic exposures in occupational settings. Further research is needed to establish standardized treatment protocols and enhance understanding of the ocular effects of palytoxin.
